# Supplementary material for: Hemojuvelin deficiency promotes liver mitochondrial dysfunction and predisposes mice to hepatocellular carcinoma
Source: Commun Biol. 2022 Feb 22;5:153. doi: 10.1038/s42003-022-03108-2 (PMC8863832; doi:10.1038/s42003-022-03108-2)
Supplement: Supplementary file 2 — Supplementary Information [file 42003_2022_3108_MOESM2_ESM.pdf]

Supplementary Figures and Tables

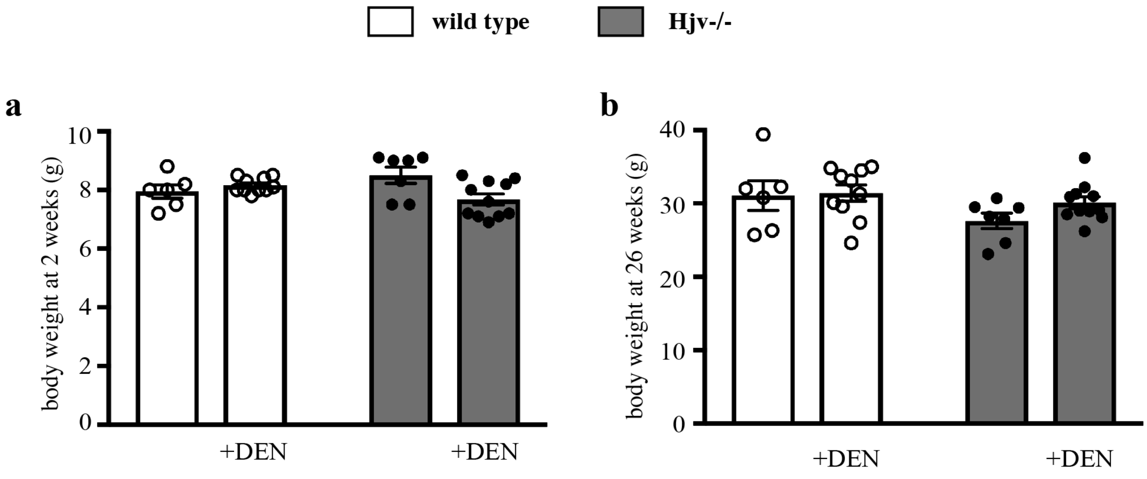

Fig. S1. Body weights of mice at the beginning (a) and at the endpoint (b) of the experiment described in Fig. 1. Data are presented as the mean ± SEM.

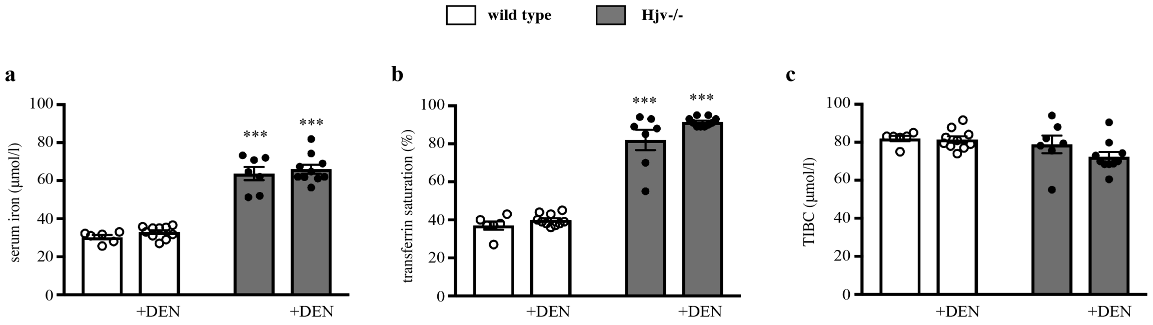

Fig. S2. Serum biochemistry data of mice at the endpoint of the experiment described in Fig. 1. (a) Serum iron. (b) Transferrin saturation. (c) Total iron-binding capacity (TIBC). Data are presented as the mean ± SEM. Statistical analysis was performed by two-way ANOVA. Statistically significant differences across genotypes are indicated by *** ($p<0.001$).

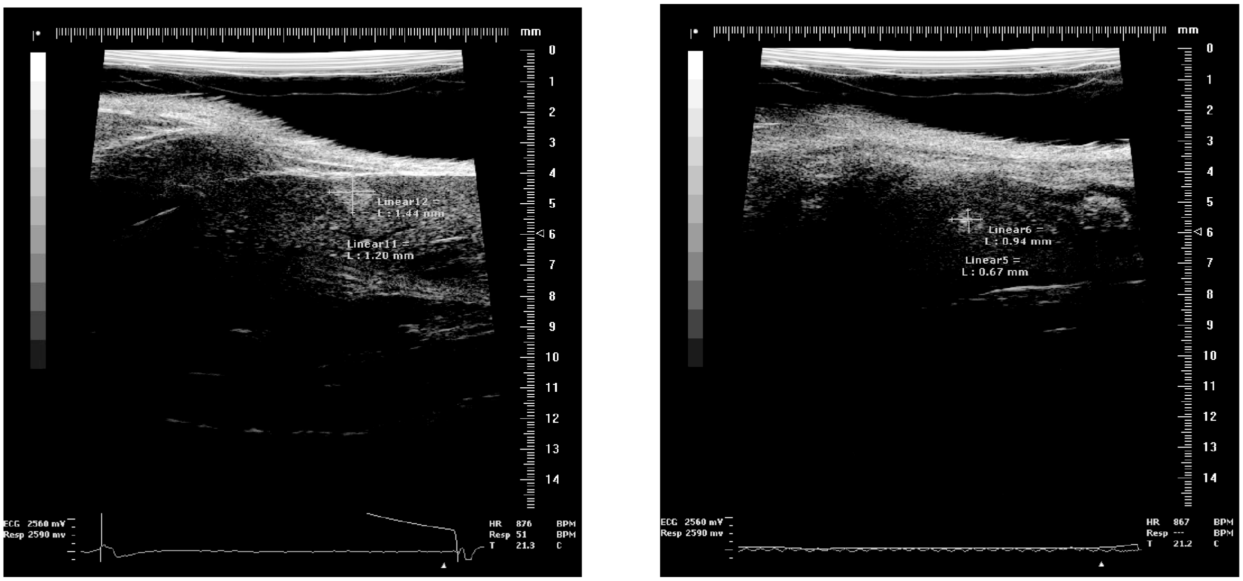

Fig. S3. Abdominal ultrasound of two representative 20-week-old Hjv-/- mice showing HCC lesions.

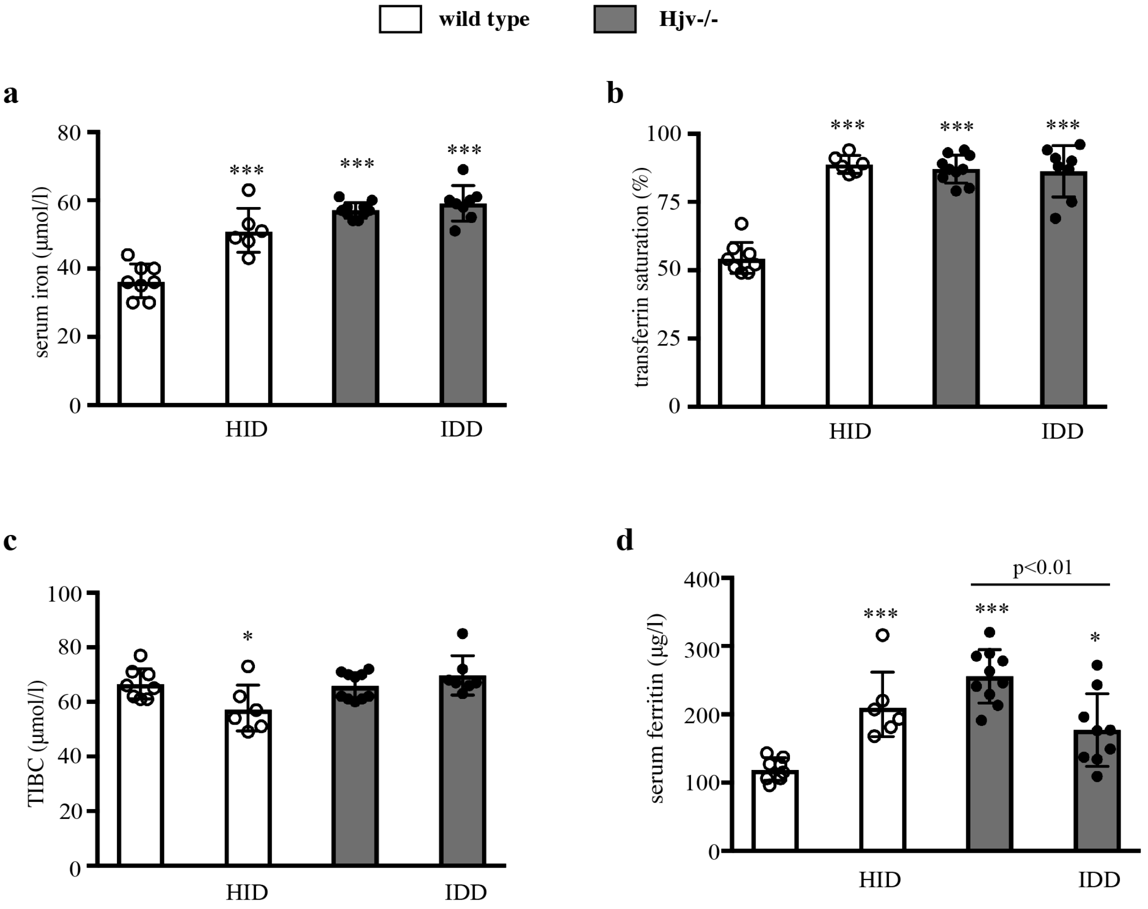

Fig. S4. Serum biochemistry data of mice at the endpoint of the experiment described in Fig. 5. (a) Serum iron. (b) Transferrin saturation. (c) Total iron-binding capacity (TIBC). (d) Serum ferritin. Data are presented as the mean ± SEM. Statistical analysis was performed by two-way ANOVA. Statistically significant differences across genotypes are indicated by * ($p<0.05$) or *** ($p<0.001$).

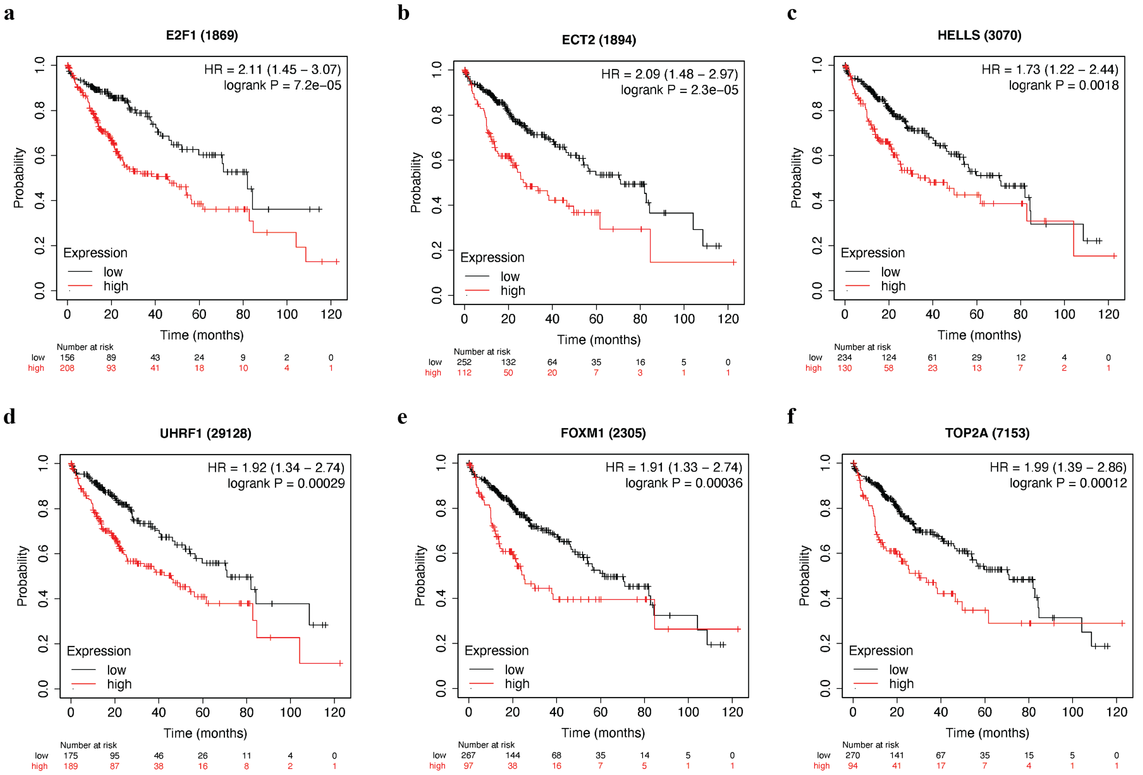

Fig. S5. High expression of oncofetal genes is associated with poor prognosis in HCC. Survival analysis in HCC patients plotted against expression of: (a) *E2F1*, (b) *ECT2*, (c) *HELLS*, (d) *UHRF1*, (e) *FOXM1* and (f) *TOP2A*. All RNAseq data were obtained from the TCGA HCC dataset.

Table S1. Spearman's rank correlation coefficients and p values of expression levels among *HAMP*, *HJV* and the oncofetal marker genes *E2F1*, *ECT2*, *FOXM1*, *TOP2A*, *HELLS* and *UHRF1*.

Spearman's rank correlation coefficients

gene	HAMP	HFE2	E2F1	ECT2	FOXM1	TOP2A	HELLS	UHRF1
**HAMP**	1	0.073	-0.168	-0.1963	-0.2286	-0.2486	-0.1428	-0.1699
**HJV**	0.073	1	-0.2799	-0.4864	-0.3874	-0.4366	-0.4284	-0.4379
**E2F1**	-0.168	-0.2799	1	0.6545	0.7763	0.7456	0.7408	0.7835
**ECT2**	-0.1963	-0.4864	0.6545	1	0.8263	0.8681	0.7598	0.8066
**FOXM1**	-0.2286	-0.3874	0.7763	0.8263	1	0.8941	0.8202	0.846
**TOP2A**	-0.2486	-0.4366	0.7456	0.8681	0.8941	1	0.8513	0.8642
**HELLS**	-0.1428	-0.4284	0.7408	0.7598	0.8202	0.8513	1	0.8318
**UHRF1**	-0.1699	-0.4379	0.7835	0.8066	0.846	0.8642	0.8318	1

p-values

	HAMP	HFE2	E2F1	ECT2	FOXM1	TOP2A	HELLS	UHRF1
**HAMP**	1.00E-04	0.1643	0.0013	2.00E-04	1.00E-04	1.00E-04	0.0063	0.0011
**HJV**	0.1643	1.00E-04	1.00E-04	1.00E-04	1.00E-04	1.00E-04	1.00E-04	1.00E-04
**E2F1**	0.0013	1.00E-04	1.00E-04	1.00E-04	1.00E-04	1.00E-04	1.00E-04	1.00E-04
**ECT2**	2.00E-04	1.00E-04	1.00E-04	1.00E-04	1.00E-04	1.00E-04	1.00E-04	1.00E-04
**FOXM1**	1.00E-04	1.00E-04	1.00E-04	1.00E-04	1.00E-04	1.00E-04	1.00E-04	1.00E-04
**TOP2A**	1.00E-04	1.00E-04	1.00E-04	1.00E-04	1.00E-04	1.00E-04	1.00E-04	1.00E-04
**HELLS**	0.0063	1.00E-04	1.00E-04	1.00E-04	1.00E-04	1.00E-04	1.00E-04	1.00E-04
**UHRF1**	0.0011	1.00E-04	1.00E-04	1.00E-04	1.00E-04	1.00E-04	1.00E-04	1.00E-04
