# Supplementary material for: Hemojuvelin deficiency promotes liver mitochondrial dysfunction and predisposes mice to hepatocellular carcinoma
Source: Commun Biol. 2022 Feb 22;5:153. doi: 10.1038/s42003-022-03108-2 (PMC8863832; doi:10.1038/s42003-022-03108-2)
Supplement: Supplementary file 3 — Description of Additional Supplementary Files [file 42003_2022_3108_MOESM3_ESM.pdf]

Description of Additional Supplementary Files

File name: Supplementary Data 1

Description: Combined quantitative protein data for the DEN and dietary iron studies.

File name: Supplementary Data 2

Description: Complete functional enrichment data of the DEN study.

File name: Supplementary Data 3

Description: Complete functional enrichment data of the dietary iron study.

File name: Supplementary Data 4

Description: Distribution of liver proteins identified in the DEN and dietary iron studies, compared to ref. 18.

File name: Supplementary Data 5

Description: Source data underlying the graphs and charts.
